# Mechanical small bowel obstruction due to appendiceal tourniquet: a case report and review of literature

**DOI:** 10.1186/s13256-019-2156-y

**Published:** 2019-08-08

**Authors:** Khaled Ahmed Ahmed, Ahmed Mohamed Farid Hamdy, Moustafa Ibrahim Seifeldin, Moustafa Refaie Elkeleny

**Affiliations:** Department of General Surgery, Alexandria Main University Hospital, Alexandria, Egypt

**Keywords:** Acute appendicitis, Appendiceal tourniquet, Mechanical small bowel obstruction, Closed loop intestinal obstruction, Appendectomy, Elderly

## Abstract

**Background:**

Acute appendicitis is known to cause intestinal obstruction. The presentation is commonly due to functional obstruction, but on very rare occasions it presents as mechanical obstruction, especially closed loop.

**Case presentation:**

We report a case of a 59-year-old Egyptian man who presented with symptoms suggestive of intestinal obstruction. On examination, he was afebrile with distended tender abdomen with no obvious hernias. There was no history of previous abdominal surgery. Laboratory investigations were within normal range except for elevated serum creatinine levels. Plain erect X-ray and computed tomography scan of his abdomen indicated mechanical small bowel obstruction.

Preoperative preparations with administration of intravenously administered fluids and antibiotics were done for exploratory laparotomy. The operation was approached through a midline incision, revealing dilated small bowel loops with a terminal ileal loop occluded by a ring of his appendix. The appendicular tip was adherent to small bowel mesentery by adhesive band (appendiceal tourniquet). Release of the band with simple appendectomy was done; a segment of ileal bowel loop was congested but regained its viability after 5 minutes’ application of gauze soaked in warm saline. His abdomen was closed in layers and one drainage tube left *in situ*. Paralytic ileus was the only postoperative complication which was relieved after 2 days. He was started on orally administered fluids on the third postoperative day, and discharged on the fifth postoperative day.

**Conclusion:**

Acute appendicitis should be suspected as a cause of mechanical intestinal obstruction in an elderly patient with no obvious diagnostic cause, and can be managed with simple appendectomy when an early intervention is made.

## Background

Intestinal obstruction is one of the most common surgical emergencies with a very wide spectrum of causes. Acute appendicitis is considered among the causes of intestinal obstruction, particularly functional obstruction. However, few cases of mechanical obstruction following acute appendicitis have been reported, with closed loop obstruction being the rare type. Closed loop obstruction may lead to strangulation and gangrene of the bowel [1]. However, its management is early intervention with simple appendectomy.

We report a rare case of closed loop small bowel obstruction due to acute appendicitis, managed by simple appendectomy through laparotomy.

We review the literature on other cases of intestinal obstruction due to appendicitis with a focus on the closed loop type. We also present a comprehensive discussion about the different presentations, diagnostic approaches, and management.

## Case presentation

A 59-year-old Egyptian man presented to our Emergency Department with a complaint of absolute constipation for 5 days associated with progressive abdominal distension and pain. Three days later, he could not tolerate any fluids or solid food and began to vomit, with no associated fever, weight loss, or previous attacks of bleeding per rectum. Also, there was no history of any medical illness or previous abdominal surgery.

On examination he was alert, afebrile, hemodynamically stable, and with no signs of dehydration. His abdomen was diffusely distended with no scars or any obvious hernias. There was lower abdominal tenderness and hyperperistalsis on auscultation. A digital rectal examination was unremarkable.

Laboratory investigations showed elevated serum creatinine level (3.9 mg/dl) whereas white blood cells count, serum sodium level, and serum potassium level were within the normal range.

A plain erect abdominal X-ray revealed multiple air fluid levels, which were suggestive of intestinal obstruction. A non-contrast abdominopelvic computed tomography (CT) scan showed small bowel obstruction with a transition zone at terminal ileum while the appendix was not well visualized.

He was started on supportive therapy with intravenously administered fluids, antibiotics, and insertion of nasogastric tube and urinary catheter.

**Fig. 1:**
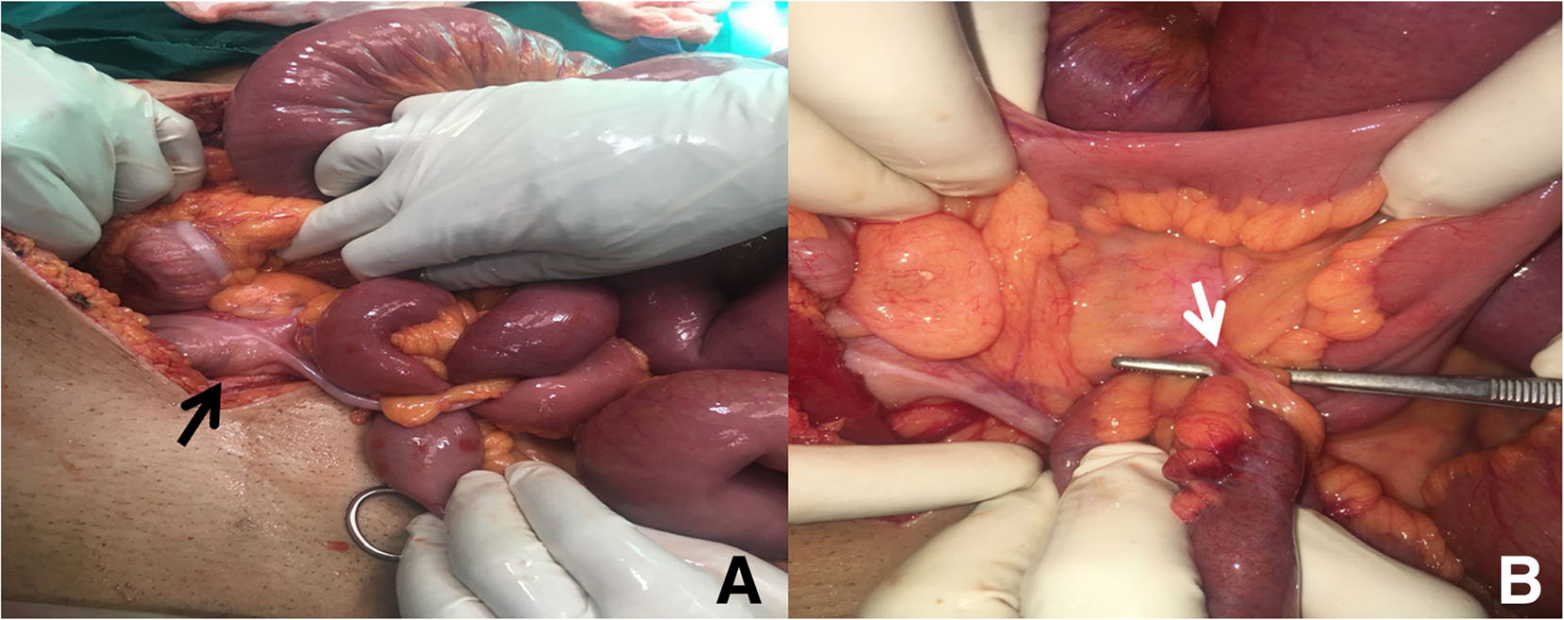
Intraoperative image. **a** Front view of the appendiceal tourniquet encircling the terminal ileal loop, base of the appendix (*black arrow*). **b** Back view of the appendiceal tourniquet, appendico-mesenteric band (*white arrow*)

A decision for exploratory laparotomy was made due to doubtful diagnosis. A midline incision was done under general anesthesia. There were dilated proximal small bowel loops to a point where a long inflamed appendix was wrapped around a loop of terminal ileum. The appendiceal tip was adherent to the ileocolic mesentery, obstructing the ileal loop at two levels causing closed loop obstruction (Fig. 1).

**Fig. 2:**
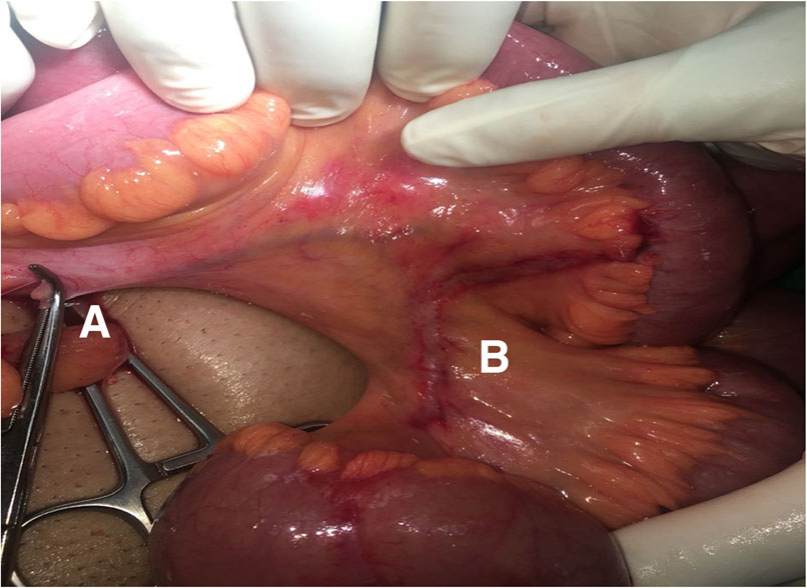
Intraoperative image. **a** Division of the adhesive band. **b** Impression mark of the appendix over the mesentery

Release of the adhesive band was achieved by separating the tip of the appendix from the mesentery (Fig. 2). The bowel loops were assessed and found to be congested, and regained their viability after 5 minutes’ application of warm saline packs. An appendectomy was done, one tube drain was inserted, and his abdomen was closed in layers. Histopathological assessment of the appendix revealed features of acute appendicitis.

He suffered from paralytic ileus for 2 days after surgery, which was managed conservatively. He was started on orally administered fluids on the third postoperative day and was discharged on the fifth postoperative day.

## Discussion

“Acute appendicitis, with its coincident peritonitis of various degrees of severity, is probably a much more common cause of acute intestinal obstruction than is generally known”; with these words, Lucius Hotchkiss introduced into the medical literature in 1901 three cases of mechanical small bowel obstruction following acute appendicitis. He emphasized mechanical small bowel obstruction rather than the functional type associated with acute appendicitis [2].

Since then, many cases of appendicitis complicated with intestinal obstruction have been recorded. As per the literature reviewed, we found that intestinal obstruction resulting from acute appendicitis is classified pathologically into two subtypes: functional or paralytic subtype and mechanical obstruction subtype.

Functional intestinal obstruction results from either: (a) inflammation, the most common type, which is due to the spread of inflammatory exudates and mediators into adjacent structures, such as the ileum and cecum [3]; or (b) ischemia, which is due to thrombosis of the ileocolic artery branches causing gangrene of the terminal ileum with loss of function, it is the rarest type [4].

Mechanical bowel obstruction may be classified into either: (a) open loop, which is due to external compression on terminal ileum by inflammatory process in the appendix, either as an abscess or appendicular mass [5]; or (b) closed loop – appendiceal tourniquet (AT). AT is a very rare condition in which the appendix is wrapped around the bowel loop; this results from adherence of the appendicular tip to the ileocolic mesentery by a band or when an inflamed appendix adheres to cecum/retroperitoneum and part of the bowel herniates through this defect [6].

The majority of the reported cases of AT were individuals of 40 years or older including this case study. This can be explained by their diminished immune response to inflammation resulting in different perceptions of pain and abnormal physical findings. The delay in diagnosis for these patients gives more time for repeated attacks of appendicitis with formation of an adhesive band between the appendix and ileocolic mesentery, especially in a lengthy appendix, which eventually obstructs the terminal ileal loop [7]. On the other hand, few cases of AT were reported in patients below 6 years of age or later in adulthood; this may be attributed to an anomalous appendico-mesenteric band [8].

AT occurs more in males than in females; this can be explained by their relatively higher pain threshold that permits repeated attacks of inflammation of the appendix with further wrapping and obstruction of the small bowel [1, 3].

The clinical presentation of this condition can be categorized as Bhandari and Mohandas (2009) reported previously into [3]:

I. Predominant features of appendicitis with some evidence of intestinal obstruction: in this group of patients, intestinal obstruction occurs during the phase of active appendicitis.
II. Predominant features of acute intestinal obstruction, while on evaluation/laparotomy found to have appendicitis as the cause. In this group, a history of appendicitis may or may not be apparent.

We observed that the picture of intestinal obstruction dominates in our case scenario and for a majority of patients with AT as well. Therefore, clinical examination is usually not typical for appendicitis, as only physical findings of intestinal obstruction are evident, while right iliac tenderness is only evident in a few cases and is usually attributed to small bowel ischemia [3, 6, 7, 9, 10].

Laboratory tests performed revealed normal potassium and sodium levels, with elevated level of serum creatinine. A leukocytes count was found to be within the normal range in our study, although it has been reported to be elevated in some series and was mainly attributed to small bowel ischemia [3, 6, 10]. Hence, leukocytosis is not a reliable indicator of acute appendicitis associated with mechanical small bowel obstruction.

Imaging tests performed in this study were non-contrast abdominal/pelvic CT scan and plain erect abdominal X-ray. The plain erect abdominal X-ray showed air fluid levels with no signs of appendicitis which was comparably the same as in other series. We performed a non-contrast CT scan of our patient’s abdomen and pelvis rather than the contrast type due to high preoperative creatinine levels, to avoid risk of contrast-induced nephropathy.

In this study, a CT scan was able to show signs of mechanical obstruction but failed to demonstrate the definite cause of obstruction. Whereas in other series, the use of a contrast-enhanced CT scan was very helpful in demonstrating obstruction, its definite cause, and the presence of bowel ischemia [3, 7, 9].

In most series, patients underwent exploratory laparotomy with diagnostic and/or therapeutic objectives. Inflamed appendix wrapped over the terminal ileum was encountered intraoperatively in almost all cases. The associated ileal loop was gangrenous in a majority of cases due to delayed presentations, necessitating appendectomy and small bowel resection as a definitive treatment [1, 9].

In this case, the bowel was found to be viable intraoperatively, as has been reported in a few series, and this may be due to early presentations of the cases seeking treatments and/or early interventions. Simple appendectomy was found to be sufficient treatment for such cases [10, 11]. Laparotomy was the commonest approach used in almost all the cases with the exception of one case that was managed laparoscopically. Further studies are needed to assess the feasibility of the laparoscopic approach in diagnosis and treatment of such conditions [12].

One mortality was reported due to postoperative sepsis [13]; however, postoperative complications of AT are usually not life threatening and can be managed conservatively, such as the paralytic ileus in our case. Other complications are wound infection and complications associated with comorbidities as reported in other cases [1, 7].

## Conclusion

Acute appendicitis may lead to mechanical small bowel obstruction, manifesting as either open or closed loop type (AT). AT occurs mainly in elderly patients with predominant features of intestinal obstruction. Examination and laboratory investigations are not helpful in indicating presence of appendicitis. Imaging may be helpful, especially contrast-enhanced abdominal CT scan.

A high index of suspicion for AT is recommended in elderly patients with mechanical intestinal obstruction with no obvious cause. Early surgical intervention prevents the bowel from becoming gangrenous, and appendectomy is sufficient to relieve the obstruction.

## Data Availability

All data generated or analyzed during this study are included in this published article.

## Abbreviations

AT: Appendiceal tourniquet
CT: Computed tomography
